# Interactions with Locust Bean Gum Regulate the Gelling Capacity of Myofibrillar Protein Emulsion: Effects of Locust Bean Gum Concentration

**DOI:** 10.3390/foods15173132

**Published:** 2026-09-03

**Authors:** Ang Ru, Xueyuan Bai, Ke Wang, Feng Yin, Xinghui Wang, Chaozhi Zhu, Gaiming Zhao, Lin Tong, Guangxing Han

**Affiliations:** 1 College of Food Science and Technology, Henan Agricultural University, Zhengzhou 450002, China; 13838575072@163.com (A.R.);; 2 Henan Key Lab of Meat Processing and Quality Safety Control, Henan Agricultural University, Zhengzhou 450002, China; 3 National Beef Cattle and Yak Industry Technology System Tongliao Comprehensive Experimental Station, Tongliao 028000, China; 4 National Beef Cattle and Yak Industry Technology System Linyi Comprehensive Experimental Station, Linyi 276000, China

**Keywords:** locust bean gum, myofibrillar protein, emulsion gel properties, gel mechanism

## Abstract

Myofibrillar protein (MP) emulsion systems tend to be unstable, leading to loss of water and oil from the emulsion gel. Thus, improvements in MP emulsification ability are needed to enhance the quality of meat products. The effects of different added concentrations of locust bean gum (LBG) (0–5 mg/g) on the emulsion gel performance of bovine MP were studied. LBG can act on myosin to stretch the spatial structure of the protein and thus improve the thermal stability of the emulsion. LBG can enhance the hydrophobicity of the MP emulsion gel molecules, allowing greater combination of the oil phase with protein, optimizing the protein’s secondary structure, and promoting greater order and uniformity in the network structure of the MP emulsion gel. LBG imparts better texture characteristics and water retention properties to MP emulsion gels. Compared with a control group, adding 5 mg/g LBG can increase the hardness of an MP emulsion from 23.11 g to 65.91 g, springiness from 0.74 to 0.82, and water holding capacity from 59.45% to 93.24%. This study provides a reference for improving the quality of beef protein-based food emulsion gel.

## 1. Introduction

Emulsified meat products are widely accepted by consumers due to their unique flavor, taste, and texture. Beef is high in protein and low in fat, and it represents the main source of meat protein in such products. Unfortunately, emulsified beef products have disadvantages such as loose texture and loss of water and oil. These issues primarily stem from the instability of the emulsified gel system, in which myofibrillar protein (MP) serves as the main structural component responsible for forming a three-dimensional gel network that immobilizes water and fat. Any breakdown of this network, whether due to weak protein–protein crosslinking, harsh processing conditions, or environmental perturbations, tends to induce phase separation and diminish the overall quality of the product. Therefore, stabilizing the gel structure of beef MP emulsions is critical for improving product quality.

In response to these problems, existing studies have offered several solutions, such as the improvement and optimization of various processing parameters, including cutting parameters [1,2]. The use of physical effects such as high pressure [3] and ultrasonic treatment [4] also has a significant effect on emulsified beef products. Adding food additives such as β-glucan [5], bacterial exopolysaccharide [6], carrageenan and pectin [7], whey protein [8], sugar beet cellulose [9], and potato starch and modified potato starch [10] falls within the scope of chemical treatment, which is more suited to small-scale or specific modifications; the advantages of these modifications are more targeted and purpose-based. The addition of food hydrocolloids offers a more targeted and flexible means to modify gel properties. From an industrial perspective, hydrocolloids are a safer and more cost-effective approach, as their minute addition levels can significantly improve the final product’s quality while reducing overall production costs through enhanced yield and stability. The interactions between polysaccharides and myofibrillar proteins play a pivotal role in meat processing; they can reinforce the gel network, improve water- and fat-binding capacity, and ultimately enhance eating quality [11,12].

Locust bean gum (LBG) comes from a leguminous plant, known botanically as *Ceratonia siliqua* L., whose seed endosperm is ground to produce a white to milky white powder. Galactomannans are linear polysaccharides that consist of a β-(l–4)-mannose backbone with single d-galactopyranosyl units attached via α-(l–6) linkages as side branches. These side branches are not distributed uniformly in the main backbone chain. There are also some unsubstituted β-D-mannopyranosyl chain segments, alternating with β-d-mannopyranosyl units substituted with α-D-galactopyranosyl side branches. Carob galactomannan is one of the commercial galactomannans, along with guar gum and tara gum, and among these galactomannans, LBG has the lowest galactose content, about twenty percent. LBG generally has a mannose–galactose ratio of about 3.5. In comparison to guar gum (1.8) and tara gum (3.0), it is the highest among other commercially available galactomannans [13,14]. This gives LBG a higher viscosity [15]. The use of LBG in food seems to be an effective and safe way to control hyperlipidemia and inflammatory bowel disease [16,17]. LBG is suitable for many food applications as it provides a creamy taste. It is often added to cream cheese sauces to increase texture and spread. It is particularly useful in preventing syneresis in various foods. LBG also creates a smooth texture in the sauce. LBG’s ability to bind water makes it an excellent choice for freezing applications, such as ice cream, as it can slow down and reduce the size of ice crystal formation so that water is retained in the ice cream. LBG is also used in yogurt production [13]. Several studies have been conducted on the effects of LBG on meat. These have used LBG in combination with high pressure and CaCl_2_ or LBG combined with xanthan gum applied in pork minced-meat gel [18,19], LBG combined with potato starch and κ-carrageenan in pork/beef mixed-meat gels [20], LBG, carboxymethyl cellulose and xanthan gum used separately and in combination in ostrich minced-meat gels [21], LBG combined with guar gum in beef minced gels [22], and LBG combined with xanthan gum used in low-fat emulsified beef minced gel [23].

The rheological, emulsifying, and gel properties of MP from different sources are known to be different [24,25]; correspondingly, the optimal amount of the same food additive in different animal-derived proteins is also different. Studies on the addition of LBG to beef protein-based emulsion gel are limited, and the role of LBG in beef protein-based emulsion gel systems is not clear. Therefore, it is necessary to study the effect of LBG addition on the properties of beef emulsion gel separately and clarify the dose–effect relationship between them, which is very important. The purpose of this study was to investigate the effect of LBG on the gelation of a beef MP emulsion at 0–5 mg/g and to suggest its mechanism.

## 2. Materials and Methods

The meat samples used were the knuckle meat muscle of Nellore Cattle (Varzea, Mato Grosso, Brazil). The tender meat muscle samples were stored at −40 °C until the extraction of the MP. Soybean oil was procured from China Food Products Marketing Co., Ltd. (Zhengzhou, Henan, China). Locust bean gum (high purity) was procured from Henan Wanbang Industrial Co., Ltd. (Zhengzhou, Henan, China). All other chemicals and reagents involved in this study were of analytical grade (AR). Analytical grade reagents such as sodium azide (NaN_3_) and glutaraldehyde were purchased from Sigma-Aldrich, St. Louis, MO, USA. Potassium chloride (KCl), sodium dihydrogen phosphate (NaH_2_PO_3_), magnesium chloride (MgCl_2_), sodium hydrogen phosphate (Na_2_HPO_3_), Triton X-100, sodium chloride (NaCl) and ethylene glycol tetraacetic acid (EGTA) were purchased from Sinopharm Chemical Reagent Corp., China. Urea, ethanol, Nile Red and Nile Blue were purchased from Solarbio Chemical Reagent Corp., Beijing, China.

### 2.1. Sample Preparation

The tender meat muscle samples were thawed at 4 °C, and the connective tissue and fat tissue were removed. The samples were chopped using a meat grinder at 4 °C before the extraction of the MP.

### 2.2. MP Extraction

MP was extracted from the meat according to the method from the article by Han et al. [26], with slight modifications. All test steps were carried out in a cold storage facility at 0–4 °C. The crushed muscle tissues were mixed with four times the mass of the buffer solution (0.1 mol/L KCl, 20 mmol/L Na_2_HPO_4_-NaH_2_PO_4_, 2 mmol/L MgCl_2_, 1 mmol/L Ethylene Glycol Tetraacetic Acid, and 1 mmol/L NaN_3_, pH7.0) and homogenized at 10,000 rpm (Ultraturrax T25 mixer, IKA-Werke GmbH Co.KG, Staufen, Germany) for 30 s. After centrifugation at 1000× *g* at 4 °C for 10 min, the supernatant was discarded, and the crude MP was retained. Four times the mass of the above buffer solution containing 1 mL/L Triton X-100 was added to wash the pellet, followed by centrifugation at 1500× *g* at 4 °C for 10 min, and this procedure was repeated twice. Specifically, after these three centrifugations, the suspension was filtered through three layers of gauze to remove connective tissue. Then, the myofibril pellet was washed with four volumes of 0.1 mol/L KCl in deionized water using the same conditions as the previously described method. The resultant MP was stored at −80 °C until further analysis. The protein concentrations of the MP were measured using bovine serum albumin (BSA, Solarbio, Beijing, China) as standard.

### 2.3. Preparation of MP/LBG Mixture

The extracted MP material was diluted to a protein concentration of 60 mg/mL with 20 mmol/L Na_2_HPO_4_-NaH_2_PO_4_, pH 7.0. Six concentrations of LBG (based on the mass of the protein solution)—0, 1, 2, 3, 4, and 5 mg/g—were added to the MP solution and then homogenized for 2 min with the Ultraturrax T25 mixer to allow sufficient distribution of the polysaccharides.

### 2.4. Preparation of MP/LBG Mixed Emulsion System

Soybean oil was added to the MP/LBG mixture (1:9, *v*/*v*) and homogenized at 10,000 rpm for 1 min using an Ultraturrax T25 mixer.

### 2.5. Measurement of the Rheological Properties of the Mixed Emulsion System

Rheological properties were analyzed using the Discovery HR-2 hybrid rheometer (TA Instruments, New Castle, DE, USA) with a parallel plate (40 mm diameter, 0.4 mm gap) geometry probe. In order to mitigate the damage to the sample network, rheological analysis was carried out in the linear viscoelastic region. The edges of the sample were covered with liquid paraffin to prevent moisture loss from the samples.

Temperature sweep measurements of the fresh emulsions containing different amounts of LBG were performed by raising the temperature from 25 °C to 80 °C at a rate of 2 °C per minute. At each temperature point, the measured conditions were 0.5% strain and a constant frequency of 0.1 Hz.

### 2.6. Differential Scanning Calorimetry (DSC) Measurement of the Mixed Emulsion System

The thermal properties of the emulsions were estimated in accordance with the method of Hwang et al. [27] using a DSC 214 (Netzsch, Selb, Germany). After diluting the emulsion to 6 mg/mL, a 2-milligram aliquot of the emulsion was taken and placed in an aluminum dish. An empty aluminum dish was used as a reference. The test temperature range was 20–100 °C. The sample was first held at 20 °C for 5 min and then heated to 100 °C at a rate of 3 °C/min. The enthalpy of denaturation (ΔH) was calculated by integrating the differential heat flow with regard to temperature, and the transition temperatures (T_max_) were assessed as the intersections of the extrapolated slopes of the peaks. The test results were measured and analyzed using Netzsch-Proteus-70 software (version 4.3) supplied by the DSC manufacturer.

### 2.7. Preparation of Gel Using Mixture Emulsion System

Soybean oil was added to the MP/LBG mixture (1:9, *v*/*v*) and homogenized at 10,000 rpm for 2 min using the Ultraturrax T25 mixer. The fresh emulsions were heated in a water bath from 20 °C to 80 °C at a rate of approximately 1 °C/min until the internal temperature reached 80 °C. After cooling to room temperature (25 °C), the next measurements were conducted.

### 2.8. Determination of the Water-Holding Capacity (WHC) of the Emulsion Gel

The WHC was determined using a centrifugal method [28]. The gel sample was centrifuged at 10,000× *g* for 10 min, and the WHC was expressed as the ratio of the weight of the gel after centrifugation to the weight of the initial gel sample. The formula is as follows:

$$\mathrm{Water}\text{-}\mathrm{holding}\;\mathrm{capacity}\left(\%\right)=\frac{M_{1}}{M_{0}}\times 100\%$$

where M_0_ is the sample quality before centrifugation, and M_1_ is the sample quality after centrifugation to remove water.

### 2.9. NMR Measurements of the Emulsion Gel

Post-treated gels were prepared as described above. Low-field NMR relaxation measurements were performed on a Niumag Benchtop Pulsed NMR Analyzer PQ001 (Niumag Electric Corporation, Shanghai, China) with a proton resonance frequency of 22 MHz. Approximately 2 g of sample was placed in the NMR tube. The spin–spin relaxation time, T_2_, was measured using the Carr–Purcell–Meiboom–Gill sequence. The T_2_ measurements were taken with a τ-value (time between 90° and 180° pulses) of 300 μs. Data from 15,000 echoes were obtained in the form of 6 scan repeats. The repetition time between subsequent scans was 8500 ms. Postprocessing of NMR T_2_ data was performed using distributed exponential fitting, and CPMG (Carr–Purcell–Meiboom–Gill) decay curves were created using Ver4.0 analysis software, which was provided by Niumag Electric Corporation.

### 2.10. Determination of the Texture of the Emulsion Gel

Each gel sample (diameter, 15 mm; height, 10 mm) was stored at 4 °C for 24 h prior to testing. Texture profile analysis of gel samples was performed using a TA. XT. Plus Texture Analyser (Stable Micro System, Godalming, Surrey, UK). A P50 cylindrical stainless-steel probe was used to compress the sample twice, with a strain–force of 5.0 g, a compression ratio of 50%, a pre-test speed of 1.0 mm/s, a test speed of 0.5 mm/s, a post-test speed of 0.5 mm/s, and a time lag of 5.0 s between the two compressions.

### 2.11. Determination of MP Intermolecular Bonds in Emulsion Gels

The chemical bonds present in the MP samples were determined according to the method from the article by Li et al. [29]. The MP samples were treated with different solutions to break specific types of bonds. The four solutions used were (A), 0.05 mol/L NaCl; (B), 0.6 mol/L NaCl; (C), 0.6 mol/L NaCl+1.5 mol/L urea; (D), 0.6 mol/L NaCl+8 mol/L urea. In short, 1 g of MP sample was added to 9 mL of each of the four buffer solutions and then homogenized for 2 min using the Ultraturrax T25 mixer. The resulting solutions were shaken for 2 min, followed by centrifugation at 8000 rpm at 4 °C for 10 min. The protein concentrations in the supernatants were measured using a Biuret assay. The amounts of chemical bonds were calculated from the differences in protein concentrations between solutions A and B (ionic bonds), B and C (hydrogen bonds), and C and D (hydrophobic bonds).

### 2.12. Raman Spectroscopy Measurement of the Emulsion Gels

Raman spectra were recorded using a Jobin Yvon LabRAM HR Evolution spectrometer (Horiba Jobi Yvon S.A.S., Longjumeau, France). The power used was about 100 mW. Frequency calibration of the instrument was performed using monocrystalline crystal silicon prior to the test, and a 50× long focal length lens was used to focus the laser on the MP emulsion gels placed on the glass slide. The spectral acquisition conditions were slit, 200 μm; 600 g·mm^−1^ grating; three scans; integration time, 60 s; resolution, 2 cm^−1^; data acquisition speed, 120 cm^−1^·min^−1^; acquisition of the Raman spectrum, 400–3600 cm^−1^. After the test was completed, the instrument’s own software, Labspec, was used to smooth the spectrum, and multi-point baseline correction was used to remove the fluorescent background. Spectra between 500 and 3200 cm^−1^ were analyzed. The stretching vibration intensity of phenylalanine at 1001 cm^−1^ does not change with the structure of the protein, so it was used as an internal standard for normalization. We used the Gaussian deconvolution fitting method in Peakfit v4.12 software (SYSTAT Software Inc., San Jose, CA, USA) to analyze the Raman spectra in the 1600 cm^−1^–1700 cm^−1^ band to analyze the protein secondary structure. The method from Alix et al. [30] was used to calculate the contents of the different secondary structures of the proteins (α-helix, β-sheet, β-turn, and random coil).

### 2.13. Observation of the Microstructure of Emulsion Gel

The microstructure of the emulsion gel before and after the addition of LBG was observed using scanning electron microscopy and confocal scanning laser microscopy.

Microstructure observation with the scanning electron microscope was performed according to the method of Cenini et al. [31] with some modifications. The sample was cut into 1 cubic centimeter cubes, fixed with a 2.5% glutaraldehyde solution, and stored at 4 °C overnight. Then, MP gel samples were rinsed three times with 0.1 M phosphoric acid buffer (pH 7.2) and subsequently subjected to gradient ethanol dehydration. Finally, the samples were dried and sprayed with gold for subsequent observation using S3400N SEM (Hitachi High-Technologies, Tokyo, Japan) with an accelerating voltage of 3 kV.

Observation using confocal scanning laser microscopy was performed according to the method of Gu et al. [32] with some modifications. The frozen emulsion gels were cut into 60 μm slices using a frozen microtome (PM2245, Leica, Nussloch, Germany), and the slices were placed on slides. A mixture of Nile red and Nile blue (0.1% and 1.0% *w*/*v*, respectively) was used to stain soybean oil and MP, respectively. After staining for 5 min, the gels were rinsed with water to remove the excess dye. The microstructures of the gels were observed using a confocal laser microscope (AX, Nikon, Tokyo, Japan).

Quantitative image analysis of all microscopic images was conducted using the ImageJ software (version 1.53). For scanning electron microscopy images, we measured the average pore diameter and porosity of the gel network; for confocal laser Raman spectroscopy images, we analyzed the average diameter of oil droplets. For each sample, three complete field-of-view images were randomly taken after sample preparation, without sub-field cropping. The Otsu automatic threshold method was used to initially segment the pore/oil droplet regions, and then the operator manually adjusted the threshold edge to ensure consistency with the contours in the original image. For oil droplets in contact with each other, the Watershed algorithm was applied for segmentation to accurately count the size of individual oil droplets.

### 2.14. Statistical Analysis

Data were expressed as means ± standard deviations from three replicates. Analysis of variance was performed using SPSS software (Version 16.0 for Windows, SPSS Inc., Chicago, IL, USA), and the Student–Newman–Keuls test was used to determine significant differences between means (*p* < 0.05). Charts were created using Origin software (Version 2018, OriginLab Corp., Northampton, MA, USA).

## 3. Results and Discussion

**Figure 1:**
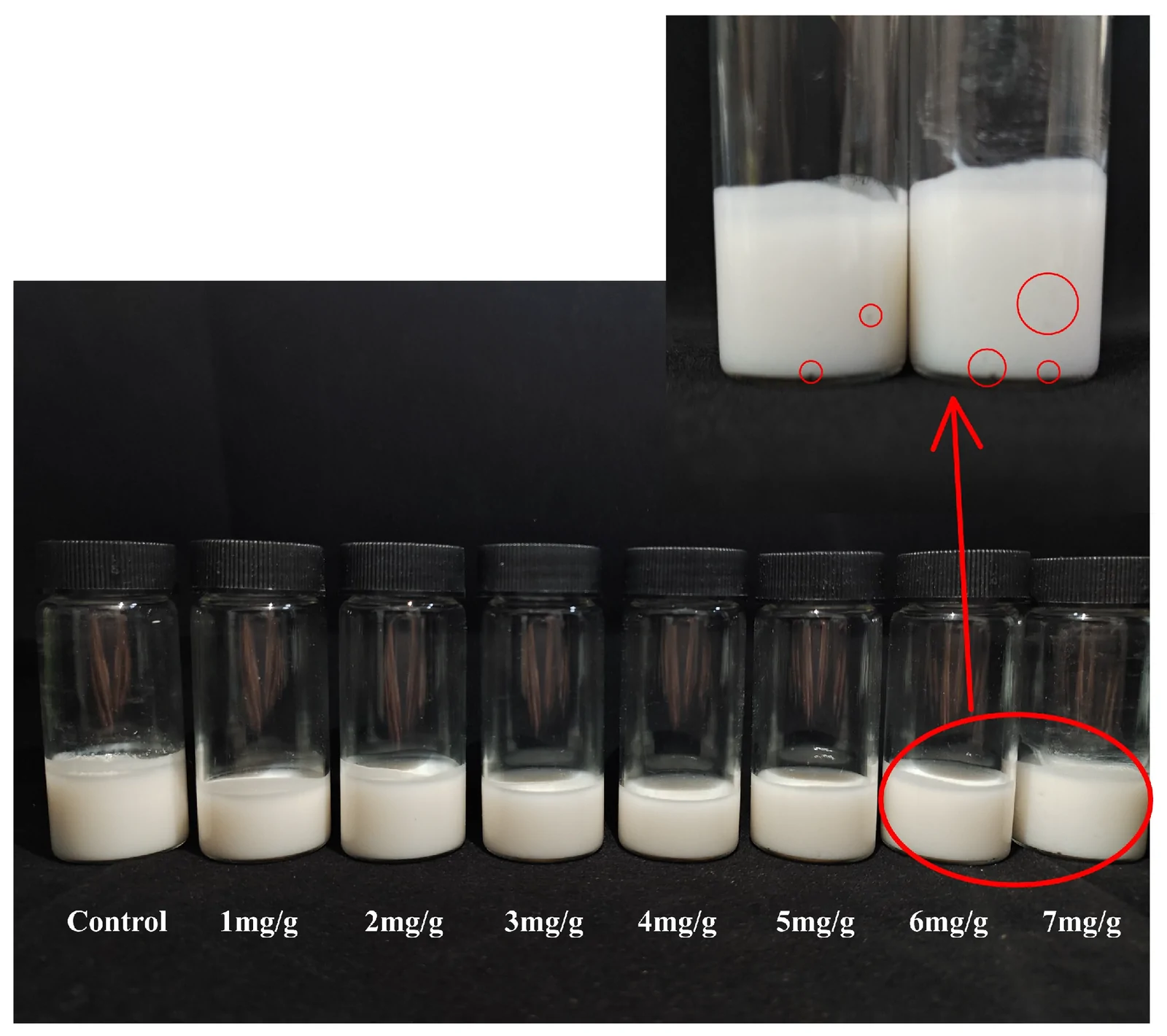
The dissolution of different amounts of LBG in MP solution. When the amount of LBG added is above 6 mg/g, LBG in the mixed system begins to become difficult to disperse evenly, as indicated by the red circle in the figure. Therefore, we set 5 mg/g as the upper limit for the addition of LBG.

As shown in Figure 1, the pre-test results showed that when the amount of LBG added to the MP solution exceeds 6 mg/g, LBG becomes difficult to dissolve in the LBG/MP mixed system; therefore, the following tests were conducted with concentrations of LBG between 0 and 5 mg/g.

### 3.1. Rheology of Mixed Emulsion System (Temperature Sweep)

**Figure 2:**
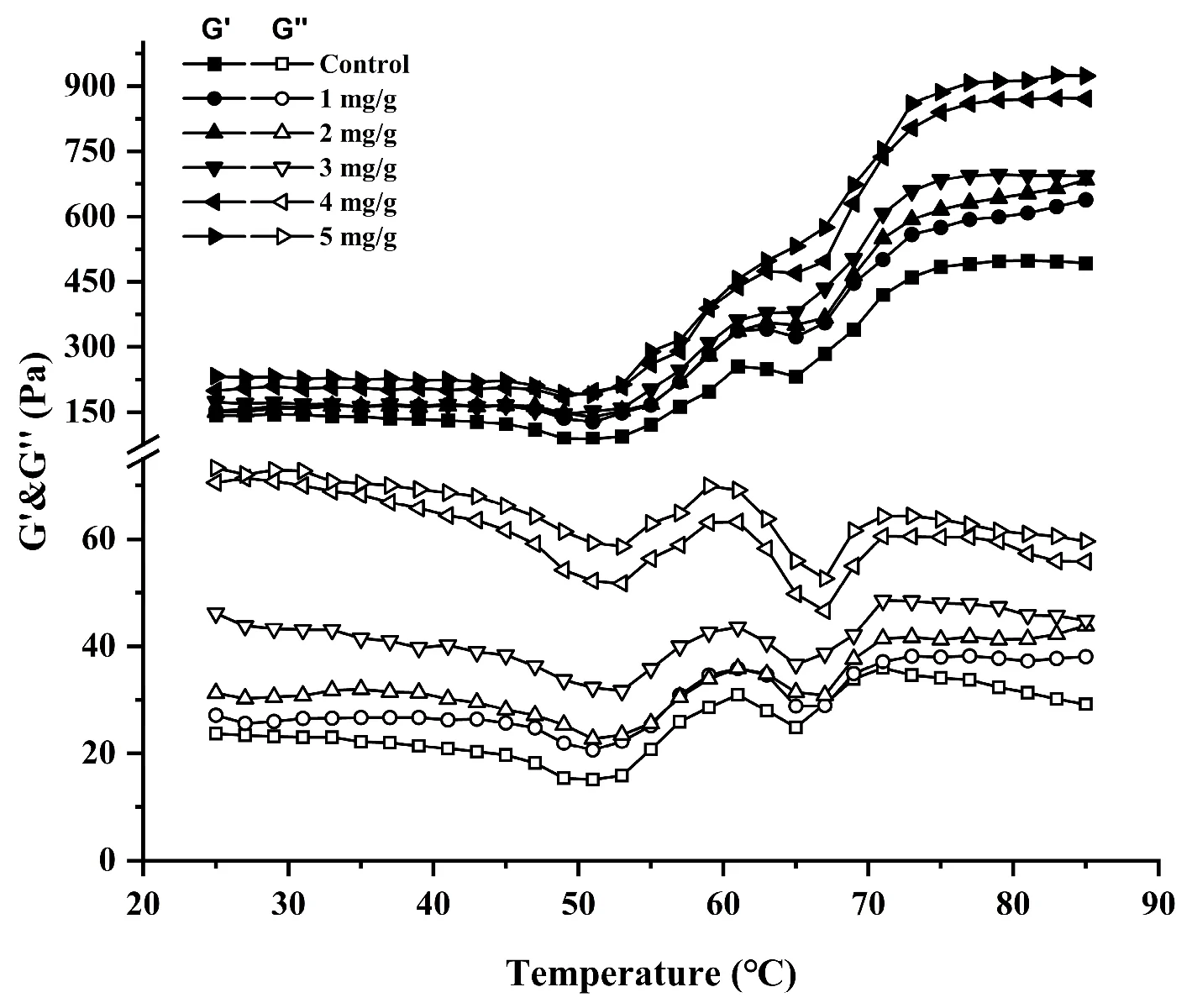
Changes in the G′ and G″ of MP emulsions after the addition of different concentrations of LBG in the temperature sweep test.

The changes in storage modulus (G′) and loss modulus (G″) in the mixed emulsion system during heating were indicative of the opening and expansion of the protein structure, which indirectly reflects the processes of protein denaturation, aggregation, and spatial network formation [25]; these results are shown in Figure 2. During the warming process, the solubilized MP undergoes an irreversible two-step rearrangement process. The first step occurs between 30 and 50 °C and includes partial denaturation and protein aggregation, especially in the large group of myosin heads. Above 50 °C, the helical light meromyosin tail groups undergo denaturation, resulting in further protein/protein interactions and leading to the formation of a three-dimensional gel structure [33]. The G′ of the emulsions without and with different concentrations of LBG showed slow and stable increases during the heating stage, with two numerical value reductions observed at around 52 °C and 65 °C. The first decrease was due to the denaturation of the myosin tails, leading to a change in fluidity and disruption of the network of meat proteins that had formed at lower temperatures. The second decrease was due to the transformation of the emulsion from a loose network to a more ordered gel matrix [34]. The position of the lost peak was shifted slightly to the right, which could indicate that the thermal stability of the MP was improved slightly by the addition of LBG. The strength of the network increased with the increase in LBG concentration, reaching the maximum G′ and G″ at a concentration of 5 mg/g. The increase in rheological moduli may be associated with an LBG-induced reduction in oil-droplet size. The smaller droplets observed under static conditions may provide a larger interfacial area for protein adsorption and crosslinking, promoting the formation of a more uniform and dense gel network during heating, which is reflected in the elevated G′ and G″ values. Furthermore, as reported in the literature, polysaccharide hydrocolloids can enhance electrostatic repulsion among protein surfaces [35], and this mechanism may also partially explain the rheological improvements observed in the present study.

### 3.2. DSC Evaluation of the Mixed Emulsion System

Protein denaturation is very important in thermal gelation. It triggers various protein–protein, hydrocolloid–protein, and fat–protein interactions that affect the gelation characteristics of MP [18,36]. We used DSC to study the temperature range of MP denaturation in the emulsion after the addition of LBG.

A previous study observed three clearly defined endothermic transitions in soluble protein at 53, 66, and 75 °C; these three temperatures are attributed to structural changes in the myosin head, myosin tail and actin, respectively [37]. Differences in muscle type, additives and pH all lead to slight differences in endothermic conversion [38], which may explain the slight differences between our test results and those reported in the literature. Table 1 shows the results of the transition temperatures (T_max_) and enthalpy of denaturation (ΔH) in MP treated with different amounts of LBG. ΔH values are expressed per gram of total emulsion sample. As the amount of LBG increased, T_max1_, T_max2,_ and ΔH increased significantly. Compared with the control group, T_max1_ increased by 1.57 °C, T_max2_ increased by 1.53 °C, and ΔH increased by 70.67% in the presence of 5 mg/g added LBG. This indicated that LBG might be bound to myosin heads and tails, which could enhance the heat stability of MP and increase the T_max_. The lack of significant change in T_max3_ meant that LBG did not have a significant effect on the heat-induced denaturation of actin. A study by Ma, Chen, Sun, Wang, Fang and Han [18] showed that the OH group of LBG may be chemically bonded to the amide I group of pork salt-soluble protein, which was consistent with our experimental results.

To sum up, the OH group of LBG might combine with the myosin amide I group during the emulsification process, such that the spatial structure of myosin becomes stretched and the internal hydrophobic group is exposed. This may reduce the interfacial tension between soybean oil and water, thereby improving the emulsifying performance of the protein. The oil-in-water system becomes more stable, and LBG can increase the viscosity of the system, thereby making the MP emulsion more stable.

### 3.3. Water-Holding Capacity and Water Distribution in Emulsion Gel

**Figure 3:**
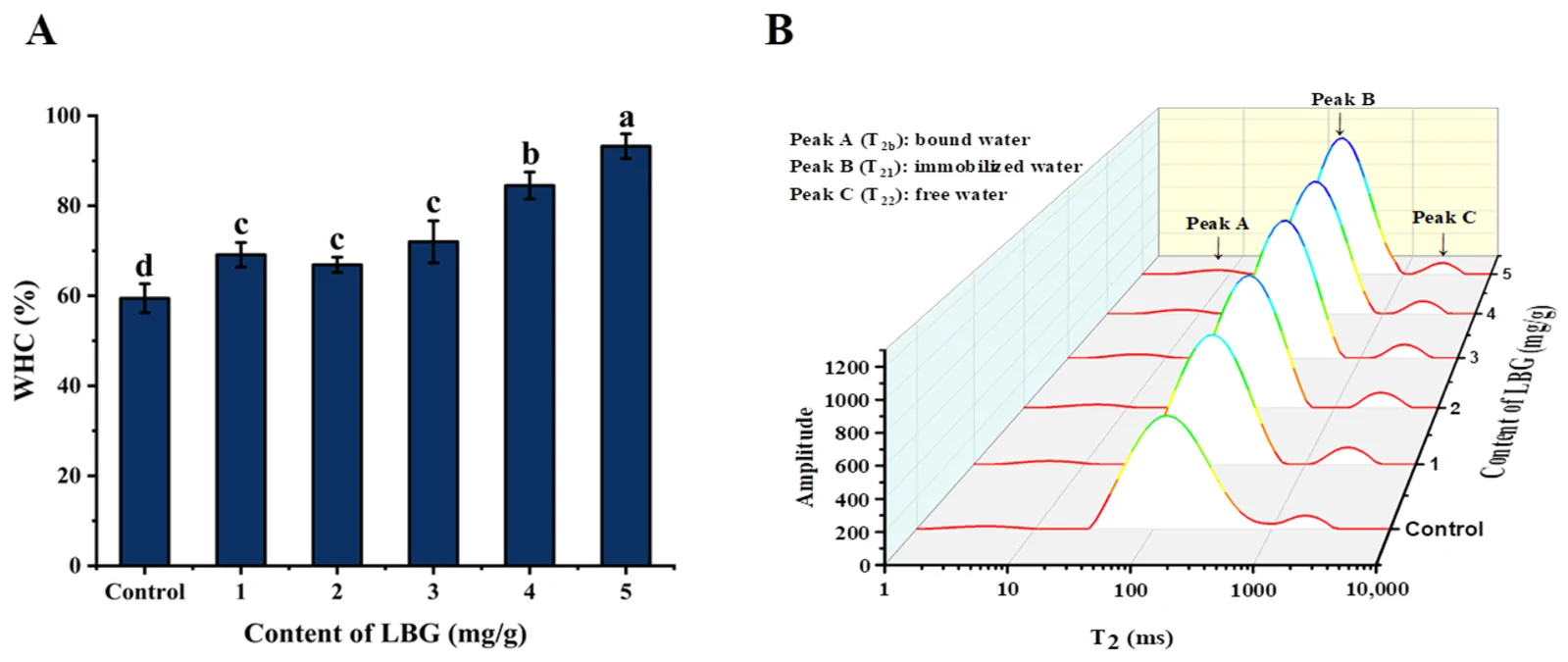
Changes in the water-holding capacity (**A**) and water distribution (**B**) of MP emulsion gels with different concentrations of LBG. In (**A**), different letters (a–d) indicate significant differences among the means (*p* < 0.05). In (**B**), the color gradation from red to blue represents the magnitude of the amplitude.

The stability of the water phase has a significant influence on the stability of the dispersed fat component and even the stability of the entire mixture in an emulsion system [33]. High WHCs are often combined with orderly and stable gel structures, binding and capturing the water network structure in three-dimensional space [39,40]. Figure 3 shows the influence of LBG on the water retention and water distribution of MP emulsion gels. Figure 3A shows that the water-holding capacity of the MP emulsion gel after LBG addition was higher than that of the control group and was best when the added amount of LBG was 5 mg/g.

The LF-NMR technique can be used to evaluate water content, distribution, and fluidity in gel networks. This technology measures the transverse relaxation time (T_2_), where a higher T_2_ value corresponds to a higher degree of water mobility, reflecting less restricted water populations within the gel network. As shown in Figure 3B, the three distinct peaks observed in MP gel represent three distinct groups of water, namely bound, immobilized, and free water, which were expressed as T_2b_, T_21_, and T_22,_ respectively [41,42], while Table 2 shows the corresponding peak area ratios for the MP emulsion gels at different LBG additions. In Figure 3B, it is clear that the amplitudes of T_2b_, T_21_ and T_22_ change as the LBG content changes. There was a significant increase in the amplitude of peak B (T_2b_), which shows that the content of bound water in the MP emulsion gel increased. The percentages of the three states of water in the MP emulsion gels are shown in Table 2. Compared with the control group, the proportion of bound water (P_2b_) and immobilized water (P_21_) in the experimental group increased, and the proportion of free water (P_22_) decreased significantly. The reason was that the addition of LBG led to greater stretching of the MP spatial structure. The increase in hydrophobic bonds between proteins stabilized the structure of the gel network, resulting in more water molecules being wrapped between proteins, leading to a reduced proportion of free water and an increased proportion of immobilized water.

Overall, the addition of LBG altered the water distribution within the MP emulsion gels, promoting the redistribution of water molecules towards a less mobile state. Specifically, the increase in immobilized water and the corresponding decrease in free water suggest that the denser and more ordered gel network physically entrapped water molecules, restricting their mobility. This redistribution is attributed to the enhanced hydrophobic interactions and the uniform network structure formed upon LBG addition, which effectively confines water within the gel matrix.

### 3.4. Texture Properties of Emulsion Gel

Texture is an important indicator for assessing the degree of protein coagulation, and is also the main parameter used to evaluate the quality of protein gel [43]. Sharp and Offer [44] showed that myosin was the most important protein in the process of forming MP thermal gels. The head-to-head aggregation, head-to-tail aggregation, and tail-to-tail aggregation of myosin molecules are the basic mechanisms involved in gel formation. During the heating process, myosin unfolds and interacts with other myosin molecules or other components to form a three-dimensional network structure [45].

**Figure 4:**
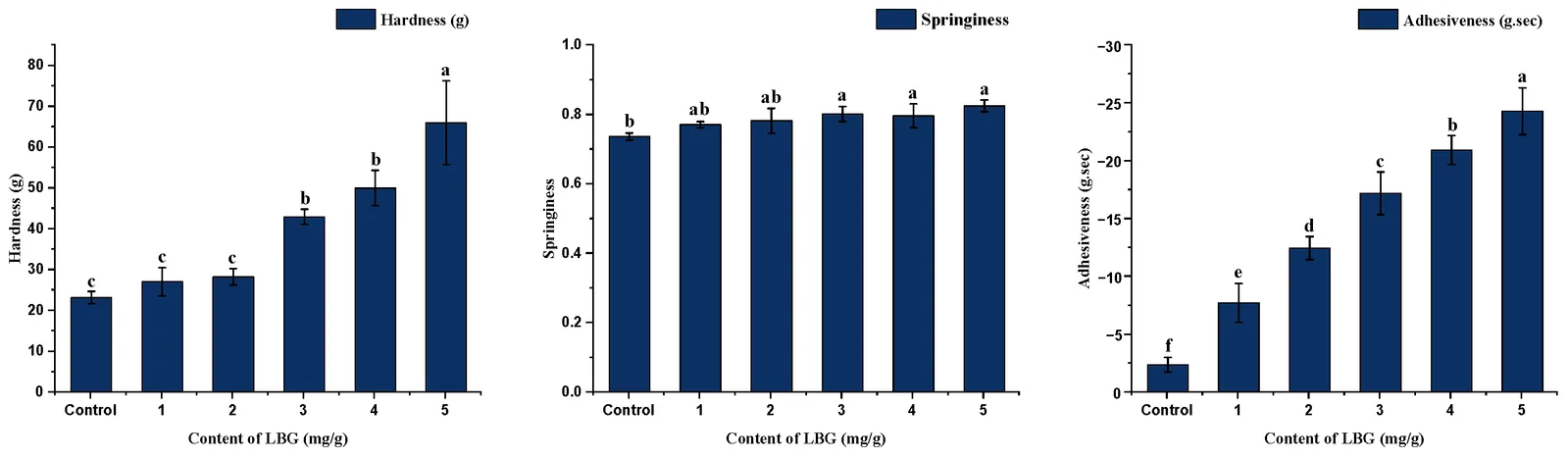
Changes in the textural properties of MP emulsion gels with different concentrations of LBG. Different letters (a–f) indicate significant differences among the means (*p* < 0.05).

In emulsion gels with active oil droplets, the interface is involved in the formation of the gel network, which can be strengthened by increasing the specific surface area [46,47]. Figure 4 shows the effect of LBG on the texture properties of emulsion gels. Greater adhesiveness, hardness, and springiness were observed in the emulsion gel system containing LBG compared to the pure emulsion MP gel (*p* < 0.05). Compared with the control group, the addition of 5 mg/g LBG increased the hardness by 185.24%, adhesiveness by 92.88%, and springiness by 12.00%. Therefore, these results indicated that the addition of LBG significantly improved the texture properties of MP emulsion gel, thereby improving the quality of emulsified beef products.

### 3.5. Raman Spectroscopy of the Emulsion Gel

**Figure 5:**
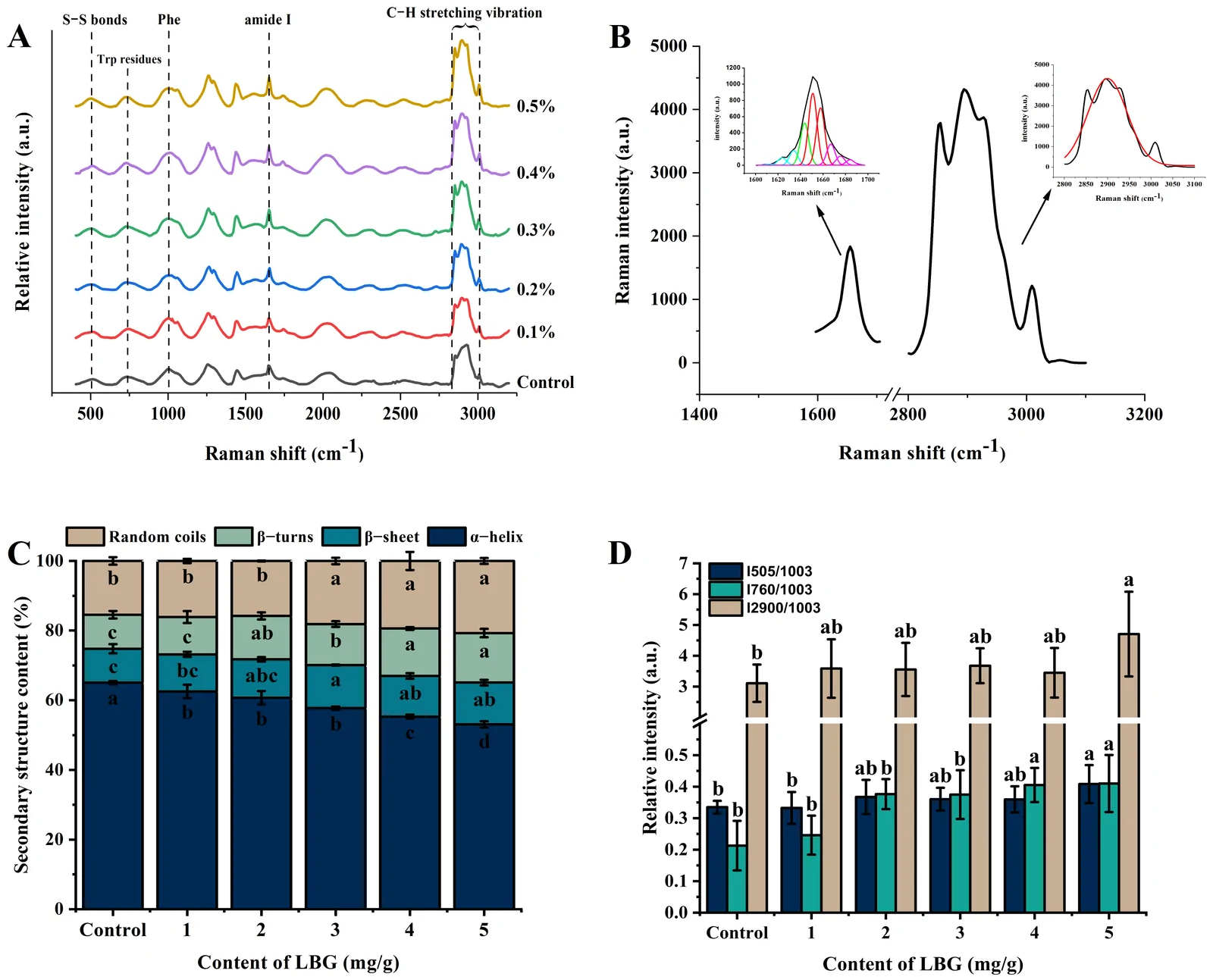
Changes in the Raman spectra and conformations of MP emulsion gels treated with different concentrations of LBG. (**A**): Changes in Raman spectra of MP emulsion gels in the range of 400–3600 cm^−1^ with different LBG contents. (**B**): Peak fitting of Raman spectra in the 1600–1700 cm^−1^ and 2800–3050 cm^−1^ bands. (**C**): Changes in the percentage of secondary structures in MP emulsion gels. (**D**): Normalized Raman spectral intensities of MP emulsion gels treated with different concentrations of LBG. In (**B**), the deconvoluted individual components are distinguished by colors: red (1650–1658 cm^−1^, α-helix), blue (1620–1640 cm^−1^, β-sheet), pink (1660–1690 cm^−1^, β-turn), and green (1640–1650 cm^−1^, random coil). In (**C**,**D**), different letters (a–d) indicate significant differences among the means (*p* < 0.05).

The properties of the MP emulsion gels were closely related to the changes in protein structure. The Raman spectra (400 cm^−1^–3600 cm^−1^) of the MP emulsion gels with varying LBG additions are shown in Figure 5A. We used the nonlinear curve-fitting Gaussian method in Origin software to fit the Raman spectra in the 2800 cm^−1^–3050 cm^−1^ band to analyze hydrophobic interactions between the protein molecules (Figure 5B).

Differences in the strength of protein gel structures were reflected in differences in the secondary structure contents of the proteins, and gel strength was found to correlate positively with the β-sheet fraction and correlate negatively with the α-helix fraction [48]. It can be seen from Figure 5C that the α-helical content decreased while the contents of β-sheets, β-turns, and disordered structures increased. Specifically, there was a significant decrease in the α-helix content from 63.92 ± 0.44% in the control group to 53.86 ± 0.90% (5 mg/g LBG group), together with a significant increase in the β-sheet content from 9.58 ± 1.29% (control group) to 12.71 ± 0.16 (3 mg/g LBG group); compared with the 3–5 mg/g groups, this difference is not significant (*p* < 0.05). In terms of β-turns and random coils, there was a significant increase from 9.59 ± 1.06% (control group) to 14.44 ± 1.23% (5 mg/g LBG group), and from 15.19% ± 1.07% (control group) to 21.02 ± 0.85% (5 mg/g LBG group), respectively. Thus, LBG could interact with myosin and rearrange intermolecular hydrogen bonds, electrostatic interactions and hydrophobic bonds within the emulsion gel, resulting in a transformation from α-helical to β-sheet, β-turn, and random coil structures. These changes in protein secondary structure help to form stronger emulsion gels.

The Raman spectra of S-S bonds near 525 cm^−1^ and tryptophan near 760 cm^−1^, as well as the C-H stretching vibration between 2800 cm^−1^ and 3050 cm^−1^, were mainly indicative of changes in the MP structure [48,49,50]. Figure 5D shows that compared with the control group, the relative strength of I505/1003 in the 5 mg/g LBG group was significantly increased, with no obvious difference in the 2–4 mg/g group. Thus, the addition of LBG helped increase the content of disulfide bonds, indicating that the cross-linking between protein cysteine residues increased, which assists in the stabilization of the tertiary structure of proteins [51], thereby improving the gelation properties of the protein [52]. The stretching vibration intensity of phenylalanine at 1001 cm^−1^ does not change with the structure of the protein, so it was used as an internal standard for normalization. The relative strength of I760/1003 increased, indicating that tryptophan residues tended to be embedded in the interior of the molecule, moving from a polar to a more hydrophobic environment [53], which was beneficial to the formation of a dense and stable protein gel network structure [50]. The relative strength of I2900/1003 increased, indicating that the surface C-H stretching vibration frequency increased, leading to exposure of internal aliphatic residues on the protein surface, which would promote surface hydrophobic interactions and enhance the interaction between protein molecules [54,55].

It can be concluded that LBG contributes to the stretching of the gel network structure, which is beneficial to the formation of an orderly gel network structure.

### 3.6. Intermolecular Forces in the Emulsion Gel

**Figure 6:**
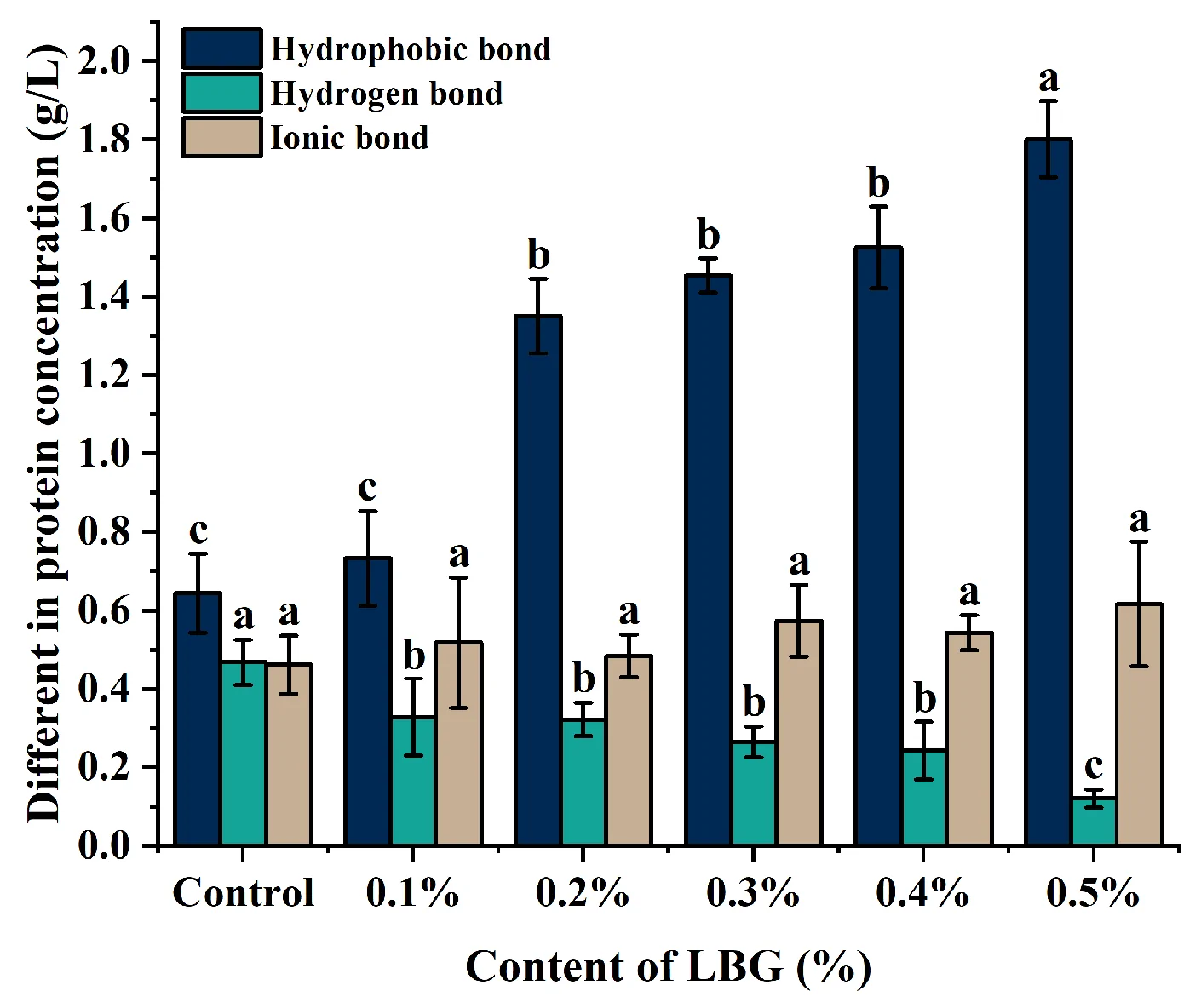
Changes in the chemical bonds of MP emulsion gels with different concentrations of LBG. Different letters (a–c) indicate significant differences among the means (*p* < 0.05).

Figure 6 shows that with the addition of LBG, hydrophobic interactions between molecules increased and hydrogen bonds were weakened, while ionic bonds did not change significantly. LBG is a non-ionic polysaccharide, so it did not contribute to ionic bonding in the MP emulsion gel [15]. Hydrophobic interactions and ionic bonds are the main contributors to the formation of the uniform dense structures of MP emulsion gels, and it has been demonstrated that enhanced hydrophobic interactions within protein gels can improve the strength of protein gels [50,56]. The addition of LBG was beneficial to the formation of hydrophobic interactions within MP emulsion gels but was not conducive to the formation and stability of hydrogen bonds. The reason is that during the emulsification of MP, LBG might caused stretching in the myosin spatial structure through the formation of hydrogen bonds with its many hydroxyl groups and amide I bands, and the hydroxyl groups occupied the original protein–protein hydrogen bond binding sites. The changes in protein spatial structure also exposed more hydrophobic groups, so that in the subsequent heating and gelation processes, more hydrophobic forces were formed to maintain the three-dimensional network structure of the MP gel. The inter-protein hydrogen bonds were thus weakened by the reduction in binding sites.

### 3.7. Microstructure of Emulsion Gel

#### 3.7.1. Scanning Electron Microscopy of Emulsion Gel

**Figure 7:**
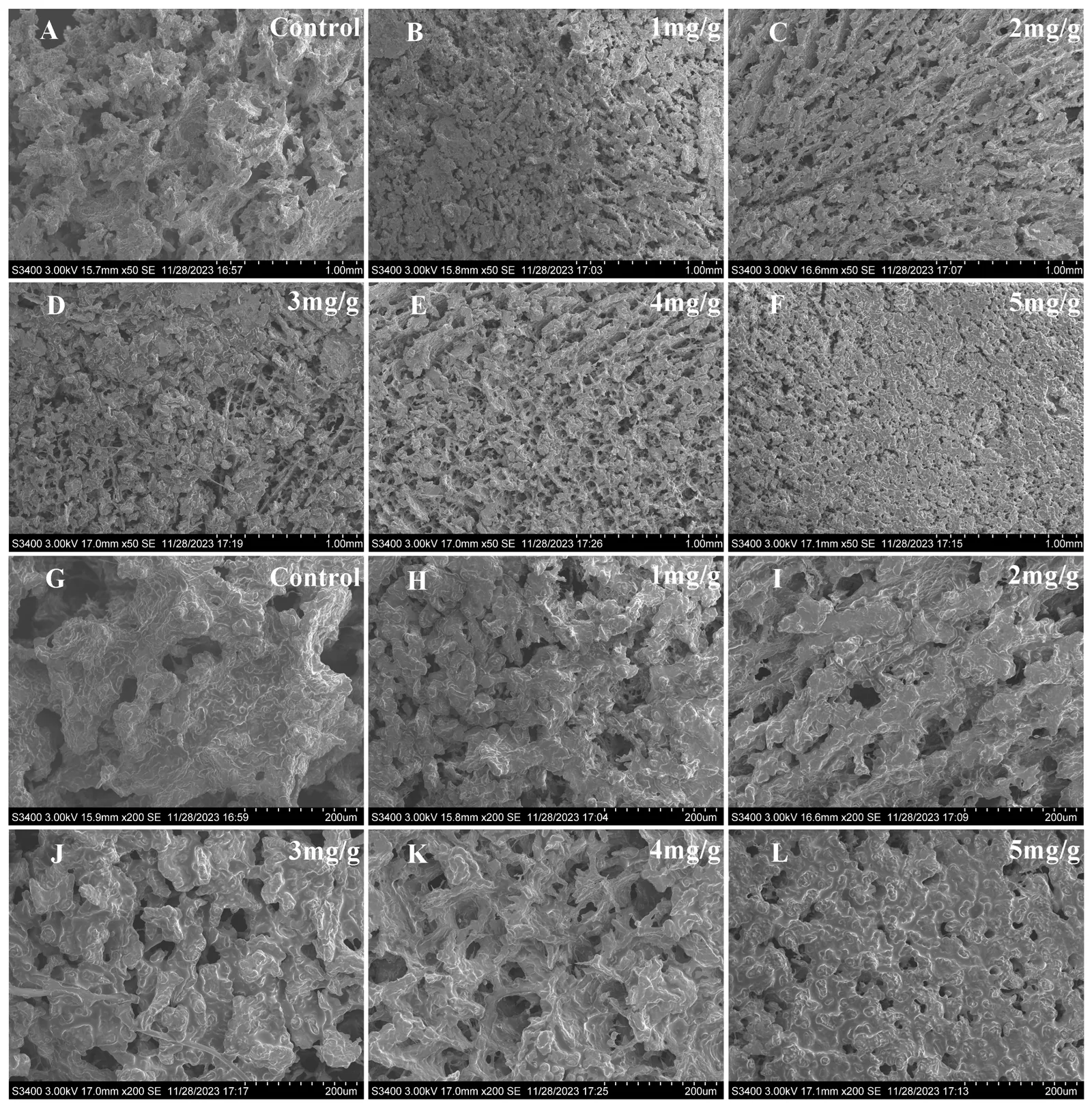
SEM microstructural images of the emulsion gels with different concentrations of LBG. Panels (**A**–**F**) are low-magnification images (×50, scale bar = 1.00 mm). Panels (**G**–**L**) are high-magnification images (×200, scale bar = 200 µm).

The microstructure and pore properties of emulsion gel after lyophilization and dehydration were observed by scanning electron microscopy [57]. As can be seen from Figure 7A,G, in the control group, the protein emulsion gel particles accumulated obviously, and the pores in the gel network were too large. After the addition of LBG, the distribution of MP emulsion gel particles became uniform, and the structure of the MP emulsion gel became dense. With the increase in added LBG, the structure of the MP emulsion gel became more stable, and the pores became smaller. Polysaccharides could stabilize the moisture content and reduce the presence of water channels in the gel network, thereby improving the formation of compact and cohesive gel networks [58]. In the control group, the network structure of the control group was irregular and divided into several clusters, and there were many “water channels”. LBG was used as a bridge in the MP structure, replacing the water channels and filling the protein network, and eventually led to a more intensive network structure, while hydrophobic bonds formed between the proteins stabilized the more intensive network structure.

To quantitatively support the microstructural observations, image analysis was performed on the SEM micrographs (Figure 7). As shown in Table 3, the average pore size of the gel network decreased significantly, and concurrently, the porosity of the gel network decreased, indicating a more compact structure.

#### 3.7.2. Confocal Laser Scanning Microscopy of Emulsion Gel

**Figure 8:**
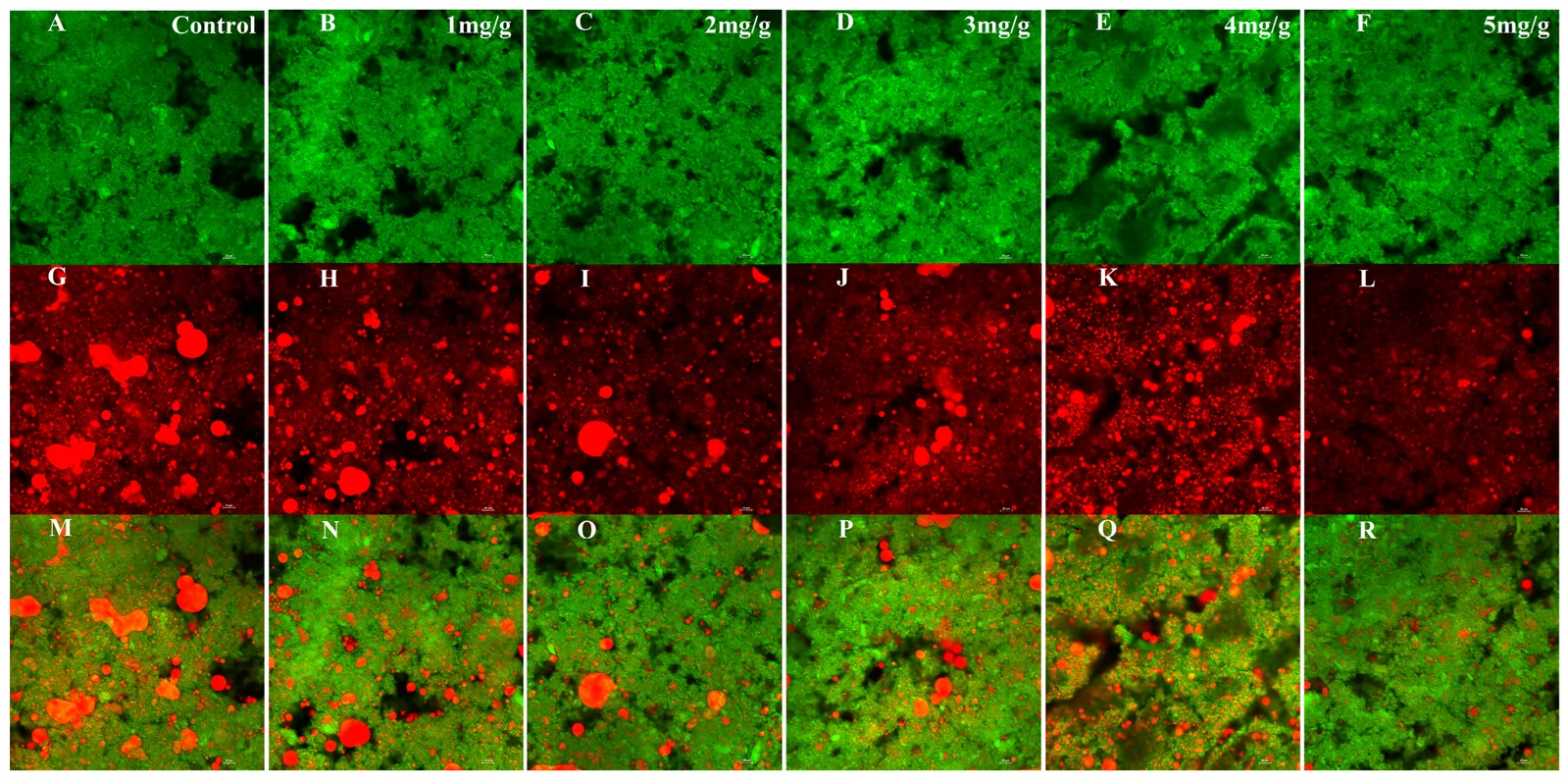
LSCM microstructure images of emulsion gels with different concentrations of LBG: (**A**–**F**) represent proteins stained by Nile Blue, (**G**–**L**) represent oils stained by Nile Red, and (**M**–**R**) represent combined images.

The distribution of fat and protein in emulsion gels was further studied by confocal laser scanning microscope (CLSM). The emulsion gel was stained with Nile Red and Nile Blue, so that the oil and protein fluoresced red and green at 520 nm and 610 nm, respectively. It can be seen in Figure 8 that when LBG is not added, the pores of the emulsion gel are thick, the network structure is loose, and the oil droplets are not evenly distributed. After the addition of LBG, a more connected and dense protein network (A–F) formed in the MP gel system, and the dispersion of peanut oil in the gel system improved. With the increase in added LBG, the dispersion effect became more obvious, and there were more and smaller oil droplets (G–L). CLSM image analysis further revealed that the mean diameter of oil droplets was reduced from 16.97 ± 2.31 μm to 3.88 ± 1.37 μm upon addition of LBG (Table 4). These quantitative results corroborate the qualitative observations and confirm that LBG promotes the formation of a denser gel network with better oil droplet stabilization.

By using SEM and CLSM, it was found that LBG could optimize the microstructure of MP emulsion gel, which was attributed to the hydrogen bond cross-linking between locust bean gum and myosin during the heating process, which promoted a change in the MP secondary structure and increased the amount of hydrophobic, hydrogen and ionic bonds among MP molecules. A more stable and dense MP network structure was formed, which effectively reduced the water loss and oil loss of the emulsion gel.

### 3.8. Proposed Mechanism for the Interaction Between LBG and MP

**Figure 9:**
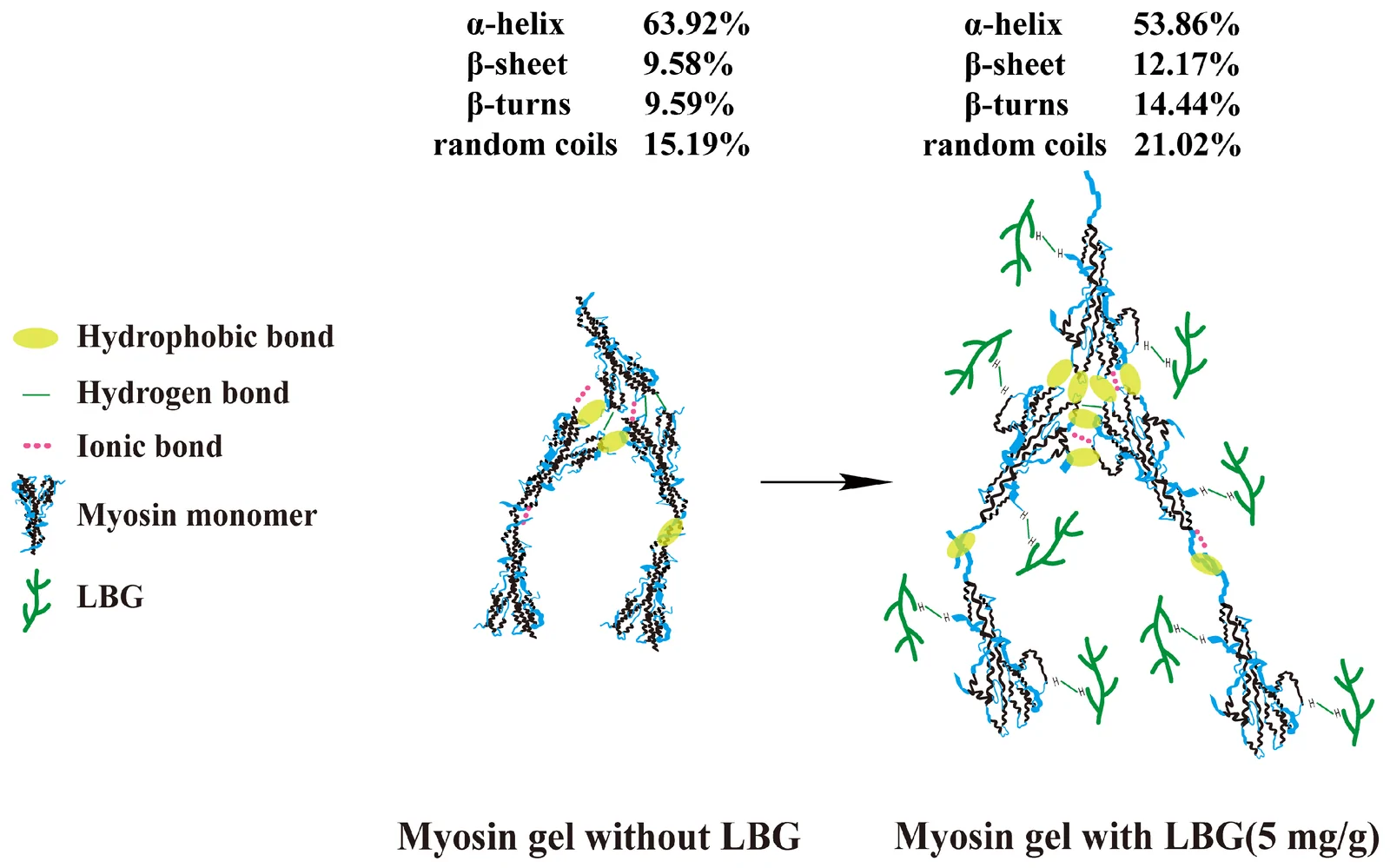
Possible interactions and changes of myosin with LBG during the gel formation process.

Considering the above results, the schematic in Figure 9 illustrates the modifications and interactions of the MP with LBG during gel formation. In the present study, we found that LBG may bind to myosin (the most important gel-forming protein in MPs) during heating gelation, and the hydrogen bonds between the LBG and protein are easier to form and stronger than those between proteins. Locust bean gum may affect the spatial structure of myosin through hydrogen bonding, exposing the hydrophobic groups within the myosin molecule to a greater extent and thus enhancing the cross-linking between proteins, ultimately improving the texture and stability of the gel.

## 4. Conclusions

In this study, the effect of LBG on the structure of MP was studied by characterizing emulsion gels. Adding LBG in the range of 0–5 mg/g can gradually improve the texture properties as well as the water and oil retention abilities of MP emulsion gel. During heat-induced gelation, LBG may stretch myosin molecules to expose hydrophobic groups within them, which can form more hydrophobic bonds, thus forming a uniform and ordered MP emulsion gel network structure. In summary, LBG can improve the gel performance and gel stability of MP emulsion gel, which provides a reference for the application of LBG in emulsified beef meat products.

## Figures and Tables

**Table 1:** Transition temperatures (T_max_) and enthalpy of denaturation (ΔH) in MP emulsions treated with LBG.

Content of LBG (mg/g)	Transition Temperatures (°C)			ΔH (J/g)
	T_max1_	T_max2_	T_max3_	
Control	56.70 ± 0.10 ^a^	65.10 ± 0.10 ^a^	73.33 ± 0.12 ^a^	0.0450 ± 0.0041 ^a^
1	56.87 ± 0.06 ^a^	66.13 ± 0.21 ^b^	73.36 ± 0.05 ^a^	0.0568 ± 0.0058 ^b^
2	57.11 ± 0.17 ^b^	66.23 ± 0.06 ^bc^	73.39 ± 0.10 ^a^	0.0605 ± 0.0013 ^b^
3	57.63 ± 0.15 ^c^	66.40 ± 0.10 ^cd^	73.43 ± 0.12 ^a^	0.0633 ± 0.0007 ^bc^
4	58.03 ± 0.06 ^d^	66.60 ± 0.05 ^d^	73.42 ± 0.03 ^a^	0.0706 ± 0.0025 ^cd^
5	58.27 ± 0.12 ^e^	66.63 ± 0.08 ^d^	73.38 ± 0.10 ^a^	0.0768 ± 0.0068 ^d^

All values are the mean ± standard deviation of three replicates. Different letters (a–e) indicate significant differences among the means (*p* < 0.05).

**Table 2:** Percentages of the three states of water in MP emulsion gels treated with LBG.

Content of LBG (mg/g)	Percentages of Three States of Water (%)		
	P_2b_	P_21_	P_22_
Control	1.49 ± 0.03 ^d^	90.67 ± 0.31 ^c^	7.84 ± 0.28 ^a^
1	1.81 ± 0.07 ^c^	91.79 ± 0.44 ^b^	6.40 ± 0.37 ^b^
2	1.88 ± 0.08 ^bc^	92.19 ± 0.60 ^b^	5.93 ± 0.53 ^b^
3	1.95 ± 0.03 ^ab^	93.35 ± 0.45 ^a^	4.91 ± 0.47 ^c^
4	1.98 ± 0.06 ^ab^	93.29 ± 0.58 ^a^	4.73 ± 0.64 ^c^
5	2.07 ± 0.07 ^a^	94.08 ± 0.13 ^a^	3.85 ± 0.19 ^d^

All values are the mean ± standard deviation of three replicates. Different letters (a–d) indicate significant differences among the means (*p* < 0.05).

**Table 3:** Image analysis of SEM microstructures of MP emulsion gels with different LBG concentrations.

Content of LBG (mg/g)	Low Magnification (×50)		High Magnification (×200)	
	Porosity (%)	Average Pore Size (µm)	Porosity (%)	Average Pore Size (µm)
Control	20.79 ± 1.68 ^a^	288.17 ± 19.36 ^a^	13.04 ± 1.57 ^a^	62.70 ± 10.89 ^a^
1	16.02 ± 1.27 ^b^	143.81 ± 17.20 ^b^	11.75 ± 1.41 ^ab^	44.21 ± 7.32 ^b^
2	15.32 ± 1.08 ^b^	110.22 ± 14.43 ^c^	11.05 ± 0.95 ^b^	43.52 ± 8.03 ^b^
3	12.51 ± 1.22 ^c^	87.95 ± 15.63 ^d^	6.75 ± 1.09 ^c^	35.07 ± 7.63 ^c^
4	11.99 ± 1.36 ^c^	66.36 ± 14.17 ^e^	6.58 ± 1.12 ^c^	18.31 ± 4.71 ^d^
5	7.70 ± 1.15 ^d^	58.96 ± 12.23 ^f^	3.78 ± 1.07 ^d^	7.45 ± 2.63 ^e^

Values are expressed as mean ± standard deviation (*n* = 3). Different superscript letters (a–f) within the same column indicate significant differences (*p* < 0.05). Pore size was calculated as the equivalent circle diameter (Feret diameter) using ImageJ software.

**Table 4:** Image analysis of LSCM microstructures of MP emulsion gels at different LBG concentrations.

Content of LBG (mg/g)	Average Droplet Size (µm)
Control	16.97 ± 2.31 ^a^
1	15.68 ± 1.75 ^ab^
2	13.78 ± 1.42 ^b^
3	10.96 ± 1.51 ^c^
4	7.26 ± 1.16 ^d^
5	3.88 ± 1.37 ^e^

Values are expressed as mean ± standard deviation (n = 3). Different superscript letters (a–e) within the same column indicate significant differences (*p* < 0.05). Average droplet size was calculated as the equivalent circle diameter (Feret diameter) using ImageJ software.

## Data Availability

The original contributions presented in this study are included in the article. Further inquiries can be directed to the corresponding authors.
